# Impact of Early Initiation of Antiretroviral Therapy in Patients with Acute HIV Infection in Vienna, Austria

**DOI:** 10.1371/journal.pone.0152910

**Published:** 2016-04-11

**Authors:** Sandra Herout, Mattias Mandorfer, Florian Breitenecker, Thomas Reiberger, Katharina Grabmeier-Pfistershammer, Armin Rieger, Maximilian C. Aichelburg

**Affiliations:** 1 Department of Dermatology, Division of Immunology, Allergy and Infectious Diseases (DIAID), Medical University of Vienna, Vienna, Austria; 2 Department of Internal Medicine III, Division of Gastroenterology and Hepatology, Vienna HIV & Liver Study Group, Medical University of Vienna, Vienna, Austria; University of Pittsburgh, UNITED STATES

## Abstract

**Background:**

It is unclear whether antiretroviral therapy (ART) should be initiated during acute HIV infection. Most recent data provides evidence of benefits of early ART.

**Methods:**

We retrospectively compared the clinical and immunological course of individuals with acute HIV infection, who received ART within 3 months (group A) or not (group B) after diagnosis.

**Results:**

Among the 84 individuals with acute HIV infection, 57 (68%) received ART within 3 months (A) whereas 27 (32%) did not receive ART within 3 months (B), respectively. Clinical progression to CDC stadium B or C within 5 years after the diagnosis of HIV was less common in (A) when compared to (B) (P = 0.002). After twelve months, both the mean increase in CD4+ T cell count and the mean decrease in viral load was more pronounced in (A), when compared to (B) (225 vs. 87 cells/μl; P = 0.002 and -4.19 vs. -1.14 log10 copies/mL; P<0.001). Twenty-four months after diagnosis the mean increase from baseline of CD4+ T cells was still higher in group A compared to group B (251 vs. 67 cells/μl, P = 0.004).

**Conclusions:**

Initiation of ART during acute HIV infection is associated with a lower probability of clinical progression to more advanced CDC stages and significant immunological benefits.

## Introduction

Since the introduction of the first antiretroviral substances the question when to start treatment has not been fully answered. International guidelines on the timing of antiretroviral therapy (ART) have changed recommending earlier treatment initiation due to emerging data showing beneficial effects of early ART. There is abundant data assessing the timing of ART in chronic HIV infection based on a certain threshold of CD4+ T cells. Several studies provide strong evidence that treatment initiation at a threshold of a CD4+ T cell count of <350 cells/μl improves survival and delays disease progression [1]. For patients with higher CD4+ T cell counts findings originate from observational cohort studies mainly suggesting a reduction of the risk of AIDS-defining illnesses and/or death when ART is initiated at <500 CD4+ T cells/μl [2]. Data on the clinical benefit of starting ART at >500 cells/μl had been inconclusive until recently, when two randomized studies showed that ART initiated immediately after HIV diagnosis and irrespective of the CD4+ T cell count leads to a significant reduction of morbidity and mortality [3, 4]. Based on these clinical trials the European AIDS Clinical Society (EACS) changed their guidelines from considering treatment to a strong recommendation for severe or prolonged symptomatic disease and a recommendation for asymptomatic acute HIV infection [5]. Since the US Department of Health and Human Services DHHS guidelines recommended ART for all HIV-infected individuals due to its effectiveness in preventing HIV transmission [6], the guidelines remained basically the same, but the strength of the recommendation changed from moderate to strong [7, 8].

Despite growing evidence of benefits of early initiation of ART, it is not clear whether treatment should be initiated during asymptomatic acute HIV infection. Several studies assessing ART during acute HIV infection show beneficial effects on laboratory progression markers. Potential positive effects of initiation of ART during acute HIV infection include reduction of viremia [9], lower viral set point [10], lower probability of transmission [6, 11, 12] and a reduced number of infected cells limiting the size of the latent pool of HIV-1 infected CD4+ T cells [13, 14]. Moreover, it has been shown that starting ART in the initial phase of infection might allow for the preservation of HIV-specific immune responses by avoiding the early destruction of CD4+ T cells and preserving the ability to control viral replication [15].

Furthermore, early detection and treatment of acute HIV infection is fundamental to avoid transmission in this critical period of high viremia and thus, high infectivity [6]. However, diagnosis of acute HIV infection is challenging, as in most cases clinical appearance is unspecific and resembles other viral infection [16]. Although there seem to be public health benefits resulting from a reduced transmission risk, cost-effectiveness has not been fully proven yet and all potential beneficial effects must be balanced against the potential disadvantages of early continuous treatment. These include long-term effects of drug toxicity, development of resistance, and adverse effects on quality of life due to the longer duration of ART [17].

## Material and Methods

### Study aim

The objective of this retrospective study was to evaluate the clinical and immunological course of individuals starting or deferring ART during acute HIV infection. The primary endpoint was the CD4+ T cell count at 12 months after HIV diagnosis (absolute or relative delta CD4+). Secondary endpoints were (i) CD4+ T cell count at month 24, (ii) HIV-RNA levels at 12 and 24 months, as well as (iii) clinical progression to CDC B and C manifestations within 5 years.

### Ethics

The study was approved by the ethics committee of the Medical University of Vienna (EK number 1297/2013). The study subjects had provided written informed consent to document their data in the Austrian HIV database.

### Study setting and data collection

This study was performed at the Department of Dermatology, Medical University of Vienna. The Austrian HIV Cohort Study (AHIVCOS) database system and stored case files were used for data collection. Epidemiological characteristics including date of birth, sex and country of birth were collected for each patient. Furthermore, HIV infection characteristics such as mode of infection with HIV (homosexual, heterosexual, intravenous drug use [IVDU]), clinical symptoms at presentation (rash, pharyngitis, lymphadenopathy, fever, other), Centers for Disease Control and Prevention (CDC) stage and ART regimen.

The following immunological parameters were evaluated at the time of diagnosis and 1, 3, 6, 12 and 24 months after diagnosis: CD4+ T cell count (cells/μl), CD8+ T cell count (cells/μl), CD4+/CD8+ ratio and HIV RNA level (copies/mL).

Moreover, we assessed clinical progression to CDC B and C manifestations within 5 years. Significant treatment interruption was defined as an ART interruption of more than 1 month.

### Study population

In accordance with the SPARTAC trial [18], acute HIV infection was defined as (i) a negative HIV antibody test with a positive HIV-RNA-PCR, (ii) a positive HIV antibody test within 6 months after a negative test for HIV, (iii) positive HIV-RNA-PCR with positive p24-antigen and less than four positive bands on Western blot analysis or (iiii) clinical manifestation of acute HIV infection supported by p24-antigen positivity and less than 4 bands in Western blot analysis. Patients with incomplete (follow-up <12 months) or missing data were excluded. Patients were grouped according to ART status and the time to ART initiation. Patients who started ART within 3 months after the diagnosis were assigned to group A. Group B comprises patients in whom no ART was initiated within 3 months after diagnosis.

### Statistical analyses

Statistical analyses were performed using IBM SPSS Statistics 21 (SPSS Inc., Chicago, Illinois, USA). Continuous variables were reported as mean ± standard deviation or median (interquartile range), while categorical variables were reported as number of patients with (proportion of patients with) the certain characteristic. Student’s t-test was used for group comparisons of continuous variables when applicable. Otherwise, Mann-Whitney U test was applied. Group comparisons of categorical variables were performed using Chi squared or Fisher’s Exact test. Kaplan-Meier curves show clinical progression or death and log-rank test was used for group comparisons. A P value < 0.05 was considered as statistically significant.

## Results

### Baseline characteristics of the study participants

**Fig 1:**
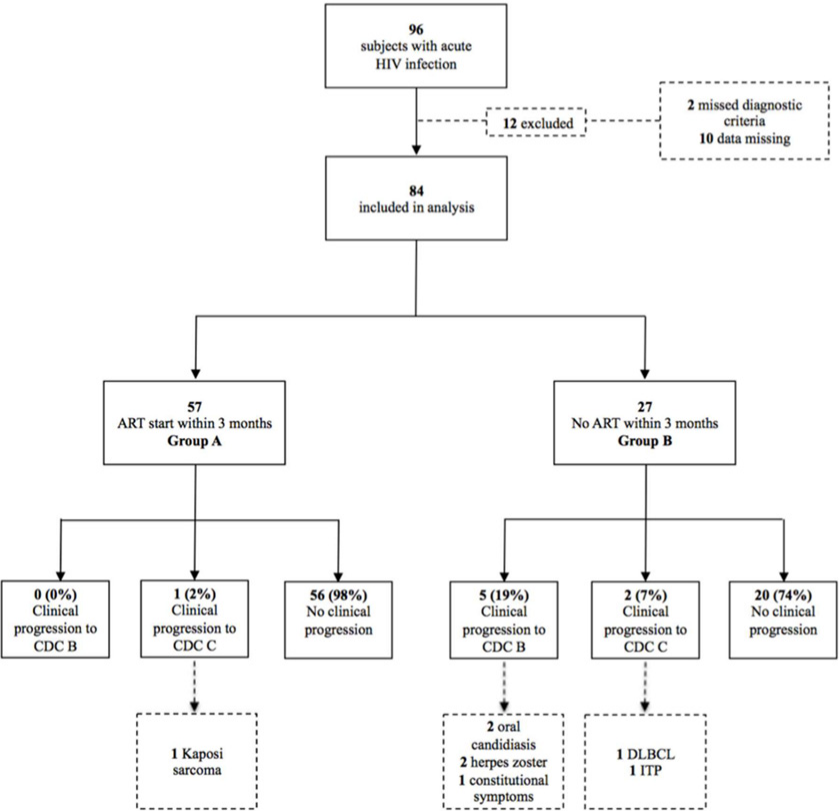
Study profile. Study profile for individuals with acute HIV infection according to treatment initiation and clinical progression during the follow up time. ART = antiretroviral therapy; CDC = Centers for Disease Control and Prevention; DLBCL = diffuse large cell B cell lymphoma; HIV = human immunodeficiency virus; ITP = idiopathic thrombytopenic purpura.

A total of 96 patients were diagnosed with acute HIV infection in the HIV clinic of the Medical University of Vienna between 1995–2013. Twelve of these patients met exclusion criteria and were thus excluded from this study. Of the remaining 84 patients, 57 (68%) received ART within 3 (group A) and 27 (32%) did not start ART within 3 months (group B) (Fig 1). The decision to start or withheld ART was solely made by the treating physician together with the patient since guidelines did not make a clear statement when to start ART in acute HIV infection.

The mean age at the time of diagnosis was 41.1 ± 9.8 years. The majority (88%, 74/84) of participants were male and 12% (10/84) were female. Eighty-two percent (69/84) of individuals were born in Western Europe, 13% (11/84) in Eastern Europe and 2% (2/84) in Africa. Transmission occurred in 18% (15/84) via heterosexual intercourse, in 69% (58/84) via homosexual intercourse and in 13% (13/84) via intravenous drug use. The majority of subjects (73%, 61/84) presented with clinical symptoms, i.e. 61% (51/84) had a maculo-papular rash, 11% (9/84) of subjects presented with pharyngitis, 32% (27/84) with lymphadenopathy and 37% (31/84) with fever. At the time of diagnosis, the median CD4+ T cell count was 427 (IQR 361) cells/μl. The median CD8+ T cell count was 1028 (IQR 816) cells/μl and the median CD4+/CD8+ ratio was 0.4 (IQR 0.43). The mean HIV viral load of the study population was 5.45 ± 1.12 log10 copies/mL (Table 1).

**Table 1:** Baseline characteristics of the study population.

	All patients		ART within 3 months, (Group A)		No ART within 3 months, (Group B)	
	n = 84		n = 57		n = 27	
**Epidemiological characteristics**						
Mean age, years	41.1	± 9.8	41.6	±10.2	40	±9.1
Sex						
Male	74	(88%)	47	(82%)	27	(100%)
Female	10	(12%)	10	(18%)	0	(0%)
Country of origin						
Western Europe	69	(82%)	45	(79%)	24	(89%)
Eastern Europe	11	(13%)	8	(14%)	3	(11%)
Africa	2	(2%)	2	(4%)	0	
Other	2	(2%)	2	(4%)	0	
**HIV infection characteristics**						
Mode of infection with HIV						
Heterosexual	15	(18%)	11	(19%)	4	(15%)
Homosexual	58	(69%)	40	(70%)	18	(67%)
Intravenous drug use (IVDU)	11	(13%)	6	(11%)	5	(19%)
Clinical symptoms at presentation						
Any	61	(73%)	39	(68%)	22	(81%)
Rash	51	(61%)	21	(37%)	21	(78%)
Pharyngitis	9	(11%)	7	(12%)	2	(7%)
Lymphadenopathy	27	(32%)	13	(23%)	14	(37%)
Fever	31	(37%)	22	(39%)	9	(33%)
CD4+ T cell count, cells/μl	427	(361)	520	(421)	414	(267)
CD8+ T cell count, cells/μl*	1028	(816)	982	(745)	1199	(1660)
CD4+/CD8+ ratio*	0.4	(0.43)	0.465	(0.47)	0.3	(0.31)
log_10_ HIV-RNA, copies/mL	5.45	± 1.12	5.4	±1.22	5.48	±0.69

* There were significant differences in baseline CD8+ T cell counts (A vs. B, *P* = 0.046) and CD4+/CD8+-ratio (A vs. B, *P =* 0.006) between the groups.

CDC = Centers for Disease Control and Prevention; HIV = human immunodeficiency virus.

### Clinical progression

**Fig 2:**
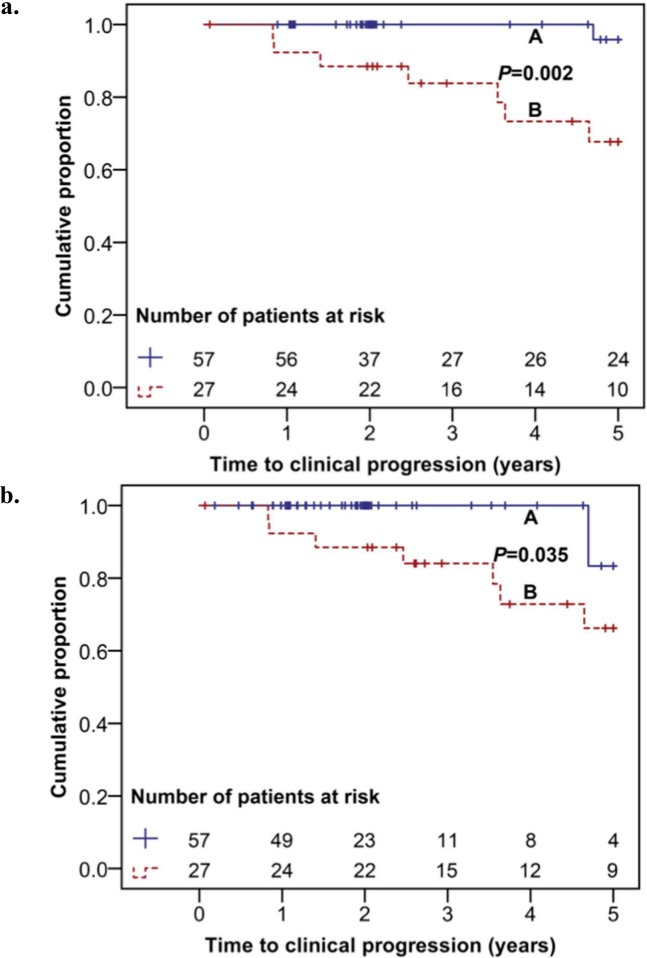
Clinical progression. (a) Kaplan-Meier curve showing the time to progression to CDC B or C manifestations for group A (treatment initiation within 3 months after diagnosis) and group B (no treatment within 12 months). (b) Kaplan-Meier curve showing the time to progression to CDC B or C manifestations for group A (treatment initiation within 3 months after diagnosis) and B (no treatment within 12 months) censoring patients with a significant treatment interruption.

The median follow-up time was 38.8 (70.3) months. Among 84 patients, 8 patients (10%) developed CDC B or C manifestations within 5 years after diagnosis. Early ART initiation was associated with a lower frequency of clinical progression (A vs. B, *P* = 0.002 (Fig 2A)). In group A, the cumulative proportion of patients without clinical progression 5 years after diagnosis was 83%. In contrast, in group B, the proportion of patients without clinical progression was only 66%. In order to account for ART interruptions, we performed an additional analysis in which patients were censored at the time of significant treatment interruptions. Similarly, the cumulative proportion of patients without clinical progression within 5 years after diagnosis were 83% and 66% in group A and B, respectively (A vs. B, *P* = 0.035 (Fig 2B)).

### Cases of CDC B and C manifestation during follow-up

The characteristics of the 8 cases of CDC stage B or C-defining illness during the observational time are shown in Table 2. The mean CD4+ T cell count was 481 cells/μl at the time of CDC B or C manifestations. Six patients had a progression to CDC stage B which included two cases of oropharyngeal candidiasis, two cases of herpes zoster and one case of constitutional symptoms and idiopathic thrombocytopenic purpura (ITP), respectively. Two patients developed AIDS, one of these individuals was diagnosed with Kaposi sarcoma and one patient developed diffuse large cell B cell lymphoma (DLBCL) (Fig 1).

**Table 2:** Characteristics of 8 cases with clinical progression to CDC B or C during follow-up.

Sex	Age (years)	Mode of infection with HIV	Baseline CD4+ T cell count (cells/μl)	Baseline viral load (copies/mL)	Time to ART (days)	ART inter-ruption	Progression to CDC stadium B or C and type of manifestation	Time to clinical progression (days)	On ART at the time of clinical progression	CD4+ T cell count (cells/μl) at the time of clinical progression	Viral load (copies/mL) at the time of clinical progression
ART WITHIN 3 MONTHS (A)											
male	37	homosexual	696	4571	9	no	C (Kaposi sarcoma)	1715	yes	unknown	unknown
NO ART WITHIN 3 MONTHS (B)											
male	57	heterosexual	417	95499	1837	no	B (oropharyngeal candidiasis)	1697	no	240	60256
male	32	homosexual	356	74131	167	no	B (oropharyngeal candidiasis)	1294	yes	959	19
male	38	heterosexual	452	630957	388	no	B (constitutional symptoms)	307	no	473	281838
male	40	homosexual	604	58884	no ART	no	B (herpes zoster)	899	no	1067	19498
male	40	homosexual	414	2754229	275	yes	C (DLBCL)	512	yes	326	49
male	30	IVDU	205	977237	1541	no	B (herpes zoster)	1327	yes	178	19
male	48	IVDU	129	758578	328	yes	B (ITP)	303	no	126	346736

ART = antiretroviral therapy; HIV = human immunodeficiency virus; IVDU = intravenous drug use; PI = protease inhibitor, NNRTI = non nucleoside reverse transcriptase inhibitor; CDC = Centers for Disease Control and Prevention; DLBCL = diffuse large cell B cell lymphoma; ITP = idiopathic thrombytopenic purpura.

At the time of clinical progression, 4 of the 8 individuals had measurable HIV RNA levels. Two of the 8 individuals with clinical progression had interrupted treatment while one had not started ART during the follow-up time.

### Comparison of individuals with or without ART within 3 months after diagnosis

All 84 study participants were included in this analysis in order to compare the immunological course of patients who received ART within 3 months (group A) with patients who did not receive ART within 3 months (group B). There was no difference between the groups in terms of age and transmission risk. Eighty-two per cent (47/57) of the patients in group A were male, whereas all patients (27/27) in group B were male (P = 0.026). All female individuals (n = 10) started ART within 3 months (group A) (Table 1).

Baseline median CD4+ T cell counts (520 (IQR 421) vs. 414 (IQR 267) cells/μl, P = 0.292) and mean HIV RNA-levels (5.4 ± 1.22 vs. 5.48 ± 0.69 log10 copies/mL, P = 0.602) were comparable among group A and B. Median baseline CD8+ T cell count was significantly lower and median CD4+/CD8+ ratio higher in group A as compared to group B (982 (IQR 745) vs. 1199 (IQR 1660) cells/μl, P = 0.048; and 0.465 (IQR 0.47) vs. 0.3 (IQR 0.31), P = 0.006) (Table 1).

### Comparison of HIV infection characteristics of individuals with or without ART within 3 months after HIV diagnosis at months 12 and 24

After one year group A showed significantly higher mean CD4+ T cell counts (721 ± 289 vs. 521 ± 249 cells/μl, P = 0.004), lower median CD8+ T cell counts (623 (513) vs. 1014 (484) cells/μl, P < 0.001) and a higher median CD4+/CD8+ ratio (1 (IQR 0.7) vs. 0.4 (IQR 0.4), P < 0.001). Median HIV RNA-levels were significantly lower in group A vs. B (1.69 (IQR 0.41) vs. 4.17 (IQR 3.1) copies/mL, P < 0.001). ART within 3 months was associated with a significantly higher increase in number of CD4+ T cells compared to deferred/no therapy (225 ± 266 vs. 87 (IQR 225) cells/μl, P = 0.002). While the changes in CD8+ T cell counts were not significantly different between the two groups (-274 (IQR 888) vs. -426 (IQR 1378), P = 0.436), the CD4+/CD8+ ratio was significantly higher in the early treatment group (0.44 (IQR 0.84) vs. 0.145 (IQR 0.33), P = 0.003) (Table 3).

**Table 3:** Comparison of individuals with ART within 3 months with individuals with no ART within 3 months twelve and twenty four months after diagnosis.

	ART within 3 months, (Group A)		No ART within 3 months, (Groups B)		*P* value
	n = 57		n = 27		
**HIV infection characteristics 12 months after diagnosis**					
CD4+ T cell count, cells/μl	721	±289	521	±249	**0.004**
CD8+ T cell count, cells/μl	623	(513)	1014	(484)	**<0.001**
CD4+/CD8+ ratio	1	(0.7)	0.4	(0.4)	**<0.001**
log_10_ HIV-RNA, copies/mL	1.69	(0.41)	4.17	(3.1)	**<0.001**
**Δ 12 months after diagnosis**					
Δ CD4+ T cell count, cells/μl	225	±266	87	(225)	**0.002**
Δ CD8+ T cell count, cells/μl	-274	(888)	-426	(1378)	0.436
Δ CD4+/CD8+ ratio	0.44	(0.84)	0.145	(0.33)	**0.003**
Δ log_10_ HIV-RNA, copies/mL	-4.19	(1.55)	-1.14	(3.02)	**<0.001**
**HIV infection characteristics 24 months after diagnosis**					
CD4+ T cell count, cells/μl	774	(400)	518	(302)	**0.001**
CD8+ T cell count, cells/μl	832	(487)	864	(437)	0.571
CD4+/CD8+ ratio	0.97	(0.64)	0.56	(0.48)	**0.002**
log_10_ HIV-RNA, copies/mL	1.43	(1.27)	3	(3.22)	0.061
**Δ 24 months after diagnosis**					
Δ CD4+ T cell count, cells/μl	251	±243	67	±284	**0.004**
Δ CD8+ T cell count, cells/μl	-126	(722)	-401	(1538)	0.103
Δ CD4+/CD8+ ratio	0.36	(0.69)	0.26	(0.55)	0.244
Δ log_10_ HIV-RNA, copies/mL	-4.01	(3.01)	-2.96	(3.56)	0.102

ART = antiretroviral therapy; HIV = human immunodeficiency virus.

Two years after diagnosis group A still showed significantly higher CD4+ T cell counts (774 (400) vs. 518 (302) cells/μl, P = 0.001), whereas the viral load was (not significantly) lower (1.43 (1.27) vs. 3 (3.22) log10 copies/ml, P = 0.061). The mean increase from baseline of CD4+ T cells was significantly higher in group A compared to group B (251 ± 243 vs. 67 ± 284 cells/μl, P = 0.004). The median CD4+/CD8+ ratio was higher in group A as compared to group B (0.97 (IQR 0.64) vs. 0.56 (IQR 0.48, P = 0.002) (Table 3).

## Discussion

The results of our study indicate that ART during acute HIV infection leads to a significant reduction of the frequency of clinical progression to a CDC stage B or C manifestation and an enhanced increase of CD4+ T cell counts in the first 2 years. Our findings suggest, that the initiation of ART during acute infection may decrease the risk of clinical progression and preserve the immune function.

The international trend of ever-earlier treatment initiation in chronic HIV infection has recently been reinforced by the unambiguous results of the START trial and the TEMPRANO trial [3, 4]. However, HIV infection is rarely detected during acute infection due to the resemblance of symptoms to other viral infections and thus, there is only limited data on the influence of treatment during this critical phase of HIV infection on the clinical course of the disease and long-term benefits. It has been postulated that initiation of ART during acute HIV infection might delay or even obviate life-long therapy as early therapy helps to preserve the immune system and boost initial host responses [16]. Recent studies found that short-course ART delays the need for initiation of long-term ART [10, 18, 19]. Moreover, 14 cases of spontaneous post-treatment controllers after interruption of prolonged ART initiated during acute HIV infection have recently been published [20]. In 2013, the SPARTAC trial showed that a 48 week-course ART started within 6 months after seroconversion delays the decrease of CD4+ T cell count below 350 cells/μl after cessation of therapy. However, this effect did not last longer than the actual duration of therapy [18]. Another observational study of Le and co-workers showed that the initiation of ART during acute HIV infection enhances the likelihood of CD4+ T cell count recovery [21]. Those findings suggest that early and prolonged therapy may lead to improvement in viral control even off therapy.

Suppression of viral replication during acute HIV infection was shown to limit viral reservoirs [22] and viral diversity [23], and reduce residual viral replication [24]. Gianella and co-workers found that (an 18-month course of) treatment started during acute HIV infection results in reduced viremia for more than 1 year after subsequent ART cessation [9]. Accordingly, van Wyl and co-workers showed that HIV-RNA levels were lower in early treated patients compared to controls. This difference however, was no longer significant after three years [25]. In the SPARTAC trial, the mean decrease in viral load was significantly higher in the group who received a 48-week treatment interval than in the untreated group at week 60 [18]. As expected, median viral loads were significantly lower in the early treated as compared to untreated group after one year in our study population. However, comparison of our study with these findings is not valid due to different study designs.

Data regarding clinical progression of HIV infection when ART is started during acute infection is limited. In our study, individuals with early treatment less frequently developed CDC B or C manifestations. Thus, our real-life data suggests, that early ART initiation during acute HIV infection might have beneficial effects on disease progression when compared to delayed ART initiation or no ART. At the time of clinical progression to CDC B or C manifestation, 4 of the 8 individuals displayed high HIV viral loads. Thus, high viral load might be associated with clinical progression of those individuals and explained by either treatment interruption or treatment failure. Therefore, a subsequent analysis was performed in order to take significant treatment interruptions into account. Interestingly, we still observed a beneficial effect of early ART.

Interestingly, all our female study participants received early ART, although it has been described that women tend to prefer a delay of ART initiation due to personal reasons [26]. A total of 69% (58/84) of study participants were men who have sex with men (MSM). This proportion is higher than the overall ratio of MSM among HIV-infected subjects in Austria (46%) [27]. We assume that higher awareness in this risk group leads to the higher proportion of MSM in our study of acute HIV infection. This finding is consistent with Casari and co-workers, who observed a higher awareness of being at risk for HIV infection among MSM, i.e. a higher probability of detection during acute infection, whereas there was a lower perception of being at risk for HIV infection among heterosexual men [28].

This study had several limitations resulting mostly from its retrospective study design: First, the follow-up time differs between individuals with 48% of the study population having less than 5 years of follow-up which makes the interpretation of clinical progression after five years difficult. Thus, we assessed clinical progression to CDC B and C manifestations only within 5 years after the diagnosis. Secondly, the groups were heterogeneous after two and five years of follow-up, as some initially untreated patients had started therapy and some “early treatment” patients may have stopped or changed their ART regimen. To investigate the long-term benefit of early treatment, the study population was too small and inhomogeneous after 5 years. As subjects with incomplete follow-up or missing data were excluded, selection bias might have been introduced towards more healthy or adherent subjects. Nevertheless, our study provides interesting information for physicians in routine clinical practice, as it compares the ‘real-life’ clinical and immunological outcome of early and delayed ART initiation in individuals treated outside of clinical trials.

In conclusion, our retrospective observational study shows that in patients who received ART within three months after being diagnosed with acute HIV infection clinical progression to CDC B or C manifestations occurred less frequently in the early treatment group during follow-up. In addition, the early treatment group showed an improved immunological course 12 and 24 months after diagnosis. However, further randomized-controlled clinical trials are required to determine the clinical benefits and disadvantages of ART initiation during acute HIV infection.

## Supporting Information

S1 File STARD Checklist. (DOC)
